# Socioeconomic Status, Frailty, and All-Cause Mortality in Korean Older Adults: A 3-Year Population-Based Prospective Study

**DOI:** 10.1155/2017/1903589

**Published:** 2017-12-14

**Authors:** Jinkyung Cho, Inhwan Lee, Soo Hyun Park, Youngyun Jin, Donghyun Kim, Ji Young Kong, Hyunsik Kang

**Affiliations:** College of Sport Science, Sungkyunkwan University, Suwon, Republic of Korea

## Abstract

**Background:**

Little is known regarding the effects of socioeconomic status (SES) and frailty on mortality in Korea.

**Objective:**

This study investigated the combined impact of low SES and frailty on all-cause mortality in Korean older adults.

**Methods:**

Study sample at baseline comprised 7,960 community-dwelling adults (56.8% women) aged 65 years and older. The Cox proportional hazards model was used to estimate the hazard ratio (HR) and 95% confidence interval (CI) of low SES and frailty for all-cause mortality.

**Results:**

Overall, low SES plus frailty resulted in an increased risk of all-cause mortality (HR = 1.56, 95% CI = 1.09–2.23, *P* = 0.015) even after adjustments for all the measured covariates, as compared with high SES plus nonfrailty (HR = 1). Among older adults aged 65–75 years, the increased mortality risk of either low SES plus nonfrailty (HR = 1.37, 95% CI = 1.02–1.84, *P* = 0.038) or high SES plus frailty (HR = 2.09, 95% CI = 1.12–3.91, *P* = 0.021) remained significant even after adjustments for all the covariates, as compared with high SES plus nonfrailty (HR = 1).

**Conclusion:**

The current findings suggest that either low SES or frailty is significantly associated with increased all-cause mortality in Korean older adults.

## 1. Introduction

Socioeconomic status (SES) is a complex construct determined by a broad spectrum of variables that is often conceptualized as a combination of financial, occupational, and educational influences. SES is a reliable predictor of morbidity and mortality [1, 2]. This finding persists across many diseases, continues across the entire lifespan, and extends across risk factors for disease [3]. A number of potential covariates may explain the link between SES and mortality, and they include poor nutrition, lack of exercise, smoking, increased number of comorbid conditions, lack of access to or underuse of health care services, and psychological factors [4, 5].

Frailty is a biological syndrome that results from cumulative declines across multiple physiologic systems and causes vulnerability to adverse outcomes such as disability, hospitalization, and death [6]. Frailty phenotype [7] and frailty index [8] are two of the most well-known operational instruments to assess this multidimensional frailty syndrome in older persons. Two instruments are not alternatives and/or substitutes because they are different and complimentary. The frailty phenotype is designed to assess the presence/absence of signs or symptoms related to five dimensions of frailty (i.e., involuntary weight loss, exhaustion, slow gait speed, poor handgrip strength, and sedentary behavior), and it serves well for the initial risk stratification of the population according to the frailty continuum (i.e., robust, prefrail, and frail). Conversely, the frailty index is composed of a long checklist of clinical conditions and diseases to assess the number of health deficits that are manifested in an individual. Therefore, the major distinctive trait of the frailty index resides in its continuous nature, although it is used to categorize dichotomous conditions of frailty (i.e., robustness versus frailty). Regardless of instruments, however, the predictive power of frailty on mortality persists across different populations and cultural contexts in both Western [9, 10] and non-Western societies [11, 12]. Several studies examined the predictive power of frailty on mortality at different age groups, reporting frailty as a robust predictor of subsequent mortality, especially later in life [13, 14].

The global population aged 60 years or older has increased in nearly all countries in conjunction with decreased mortality and declining fertility [15]. Due to the same reasons in conjunction with increasing average life expectancy at birth, Korea is moving toward an aged society at the fastest pace in the world. In 2010, Koreans aged 65 years and older reached 11% in the population, and the number is projected to increase to 24.3% in 2030 and reach 37.4% in 2050 [16]. Consequently, it is expected that many of older adults in Korea are likely to become vulnerable to low SES and/or frailty [17], contributing to increased risk of mortality in that population.

Despite the well-established predictive power of frailty for mortality and the known association of SES with mortality, no study has been conducted to investigate the combined effect of SES and frailty on mortality risk in older adults in Korea. Therefore, this study examined the effect of exposure to both low SES and frailty on 3-year cumulative, all-cause mortality using a large nationally representative sample of older adults aged 65 or older in Korea.

## 2. Methods

### 2.1. Study Sample (Data Source)

This study was conducted using data from the 2008 and 2011 Living Profiles of Older People Survey (LPOPS), which was initiated in 2008 as a national wide 3-year interval longitudinal survey. Details of the LPOPS design are described elsewhere [18, 19]. Briefly, the survey employed stratified two-stage cluster sampling. The primary sampling unit was based on the 2005 census frame, with secondary sampling units consisting of households with older residents. The strata consisted of 7 metropolitan and 18 provincial (urban and rural) regions. Sampling within secondary geographic strata employed auxiliary stratification indices, such as gender ratio and average age obtained from surveys to yield a representative sample.

The baseline survey, Wave 1, was conducted in 2008, and a follow-up survey, Wave 2, was conducted in 2011. In Wave 1, a total of 12,087 noninstitutionalized adults aged 65 and older were initially contacted and invited to participate in the baseline assessments, and 4,127 were excluded due to refusal, incompletion of the assessments, or other person reasons, leaving a total of 7,960 subjects who completed the baseline assessments (43.2% men and 56.8% women) (response rate = 65.6%). In Wave 2, 576 were additionally excluded due to refusal, hospitalization, institutionalization, or loss of contact. A total of 6,929 subjects excluding 455 deaths completed the 2011 follow-up assessments (response rate = 87.0%) and were included in the final analyses. The Sungkyunkwan University Institutional Review Board, in accordance with the World Medical Association Declaration of Helsinki, reviewed and approved the study protocol (SKKU 2017-06-009).

### 2.2. Determination of All-Cause Mortality

The primary outcome of this study was all-cause mortality, which is a robust and unbiased index that does not require adjudication to avoid biased clinical assessments or documentation [20]. Mortality was identified by death certificate filed upon death at the registry office of the municipality of residence. For the present analyses, follow-up time was defined as the period from the baseline visit till the day of death for participants who died or the last contact date for those who did not have the outcome event. Number of deaths from all causes during the 3-year follow-up period was 455 (5.7%).

### 2.3. Determination of Socioeconomic Status and Frailty Phenotype

Economic indicators such as household income and wealth are used less frequently but are potentially as important as or more important than education and occupation [21]. Sources of household income included earnings, assets, pensions, welfare transfers, private transfers, and others of study participants and their spouses. Therefore, the cutoff point of the household income for low SES in this study was defined as the gender-specific median value of the income for the 2008 calendar year limited to the study population: low SES as ≤50th percentile or high SES as >50th percentile. The median value of the household income used in this study was found to be equivalent to below 50% of the population's median income (the poverty line) for 2011 and 2012 in Korea [22]. Frailty was assessed with a modified version of the original cardiovascular health study (CHS) frailty index [7] using the following 5 components (refer to Supplementary Table 1): (1) unintentional weight loss of 5 kg or more; (2) weakness of handgrip strength, ranking in gender- and body mass index- (BMI-) specific lowest 20th percentile of the study population; (3) exhaustion, shown by answers to the center of epidemiologic studies depression (CES-D) scale questions “I felt that everything I did was an effort” and “I could not get going” that were rated “moderate amount to most of the time during the last week”; (4) slowness, ranked in gender- and height-specific lowest 20th percentile of the study population; and (5) low physical activity (in kcal) on the international physical activity questionnaire [23], ranked in gender-specific lowest 20th percentile. The subjects having a score of 0–2 were considered to be in robust or nonfrail condition, and ≥3 indicated frail condition.

### 2.4. Covariates

Height was assessed using a measuring tape with the participant standing with the back of the head, scapulae, buttocks, and heels in contact with a vertical board. Body weight was measured using a portable digital scale, after removing the shoes and wearing only light clothing. Body mass index (BMI) was calculated by dividing body weight by height squared (kg/m^2^). Sociodemographic factors including age, gender, and education were included as covariates.

Health-related factors were also measured and included alcohol consumption and smoking, nutritional status, number of comorbidities and medications, disability, cognitive impairment, and fall experience. Smoking status was categorized as nonsmoker, past smoker, or current smoker. Alcohol consumption was classified as abstinent, moderate (1-2 drinks/week), or heavy (more than the moderate level of consumption). Nutritional score was assessed using the nutrition screening initiative checklist [24]. Comorbidity was defined as number of physician-diagnosed chronic conditions (hypertension, stroke, angina, diabetes mellitus, arthritis, chronic bronchitis/emphysema, asthma, cancer, chronic renal failure, and fracture). Disability was measured using the Korean version of the Instrumental Activities of Daily Living Scale (K-IADL). Cognitive function was assessed using the Korean version of the Mini-Mental State Examination (MMSE-KC) [25]. Falls were defined as one or more falls in the past 12 months. To assess the history of falls, participants were asked whether they had fallen during the past 12 months and, if yes, how often.

### 2.5. Statistical Analyses

Sampling weights were applied to account for the complex sampling, in order to represent all older Korean adults without biased estimates. These considerations included stratification by region at the first step and stratification by sex and age at the second step. We also adjusted for age and sex to avoid bias from changes in age and sex distributions in each phase using the Korean population distribution in the year 2005.

Descriptive statistics were presented as means and standard deviations and frequencies and percentages for continuous and categorical variables, respectively. One-way ANOVA and Chi-square were used to compare mean differences in continuous and categorical variables, respectively, according to gender and SES and frailty-based subgroups. For the subgroup classifications of study participants, SES was classified as low or high according to the median values of gender-specific incomes. Frailty was defined as meeting three out of the 5 phenotypic criteria: low grip strength, low energy, slowed waking speed, low physical activity, and/or unintentional weight loss (Supplementary Table 1). The participants were classified into the following 4 subgroups based on a combination of SES status (low versus high) and frailty (frailty versus nonfrailty); high SES plus nonfrailty, low SES plus nonfrailty, high SES plus frailty, and low SES plus frailty. The Kaplan-Meier procedure with log-rank tests was used to estimate all-cause mortality functions among the SES plus frailty-based subgroups. The Cox proportional hazards model was used to estimate the hazard ratios of a combination of SES plus frailty for all-cause mortality in this study population. Covariates include age, gender, BMI, education, number of comorbidity, number of medications, health risk behaviors (i.e., alcohol consumption, smoking), nutritional status, K-IADL, cognitive function, and fall experience. All analyses were performed taking into account complex sampling weights, using SPSS-PC version 18.0 (Chicago, IL, USA).

## 3. Results

Table 1 represents the descriptive statistics of the participants in the study. With respect to demographic variables at baseline, women were older (*P* < 0.001) and heavier (*P* < 0.001) than men. Men had higher levels of income (*P* < 0.001) and education (*P* < 0.001) than women. With respect to health risk behaviors and other covariates at baseline, men had higher rates of alcohol consumption (*P* < 0.001) and smoking (*P* < 0.001) than women. Women had higher rates of dissatisfied nutritional status (*P* < 0.001), higher numbers of comorbidity (*P* < 0.001) and medications (*P* < 0.001), and higher K-IADL scores (*P* < 0.001) and fall experience (*P* < 0.001) than men. Men had higher MMSE-KC scores (*P* < 0.001) than women.

Table 2 represents the descriptive statistics of the 4 subgroups based on a combination of SES and frailty. Mean age and BMI were 71.2 ± 5.6 and 23.7 ± 3.1 kg·m^2^ for those with high SES plus nonfrailty, 72.7 ± 5.7 years and 23.5 ± 3.3 kg·m^2^ for the low SES plus nonfrailty group, 76.3 ± 7.0 years and 23.4 ± 3.9 kg·m^2^ for the high SES plus frailty group, and 76.3 ± 6.4 years and 23.0 ± 3.6 kg·m^2^ for the low SES plus frailty group. Significant differences were found in education (*P* < 0.001), alcohol consumption (*P* < 0.001), smoking (*P* = 0.001), medications (*P* < 0.001), and comorbidity (*P* < 0.001) among the subgroups. Levels of education were highest in the high SES plus nonfrailty group, followed by the low SES plus nonfrailty, high SES plus frailty, and low SES plus frailty group in order, while numbers of comorbidity and medications were highest in the low SES plus frailty group, followed by high SES plus frailty, low SES plus nonfrailty, and high SES plus nonfrailty group in order. Significant differences were in nutritional status (*P* < 0.001), K-IADL scores (*P* < 0.001), MMSE-KC scores (*P* < 0.001), and fall experience (*P* < 0.001) among the subgroups, with no such significant group differences in alcohol consumption and smoking among the groups. Satisfaction level of nutritional status and cognitive function were lowest in the low SES plus frailty group, followed by the high SES plus frailty, low SES plus nonfrailty, and high SES plus nonfrailty group in order. Fall experience was highest in the low SES plus frailty group, followed by the high SES plus frailty, low SES plus nonfrailty, and high SES plus nonfrailty group in order.

**Figure 1:**
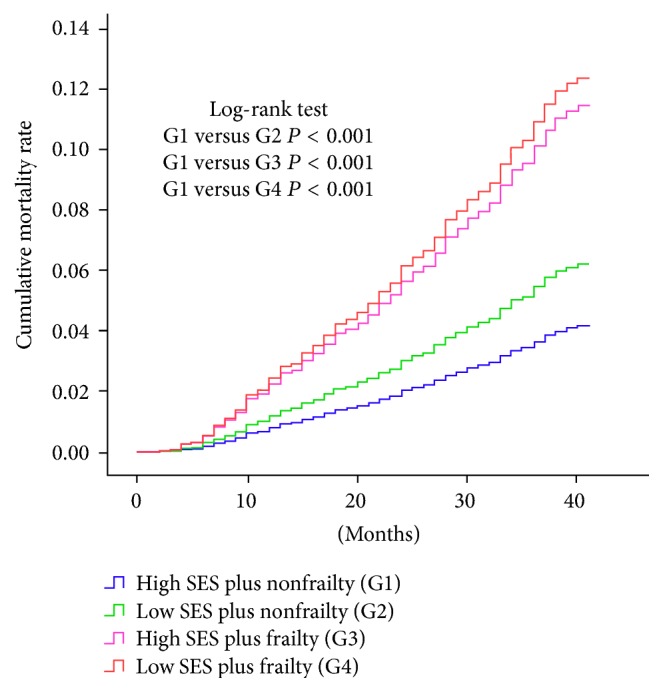
Kaplan-Maier survival curves according to socioeconomic status (SES) and frailty.

A total of 455 (5.7%) died with all causes during the 3-year follow-up period. Low SES and frailty additively contributed to increased all-cause mortality (Figure 1); their impact was highest in the low SES plus frailty group, followed by the high SES plus frailty, low SES plus nonfrailty, and high SES plus nonfrailty group in order.

Table 3 represents the hazard ratio (HR) of the 3-year-all-cause mortality according to SES and frailty status. In the entire sample, the low SES plus nonfrailty group (HR = 1.51, 95% CI = 1.23–1.85, *P* < 0.001), high SES plus frailty group (HR = 2.86, 95% CI = 1.89–4.34, *P* < 0.001), and low SES plus frailty group (HR = 3.12, 95% CI = 2.26–4.28, *P* < 0.001) had significantly higher all-cause mortality risks, as compared to the high SES plus nonfrailty group (referent, HR = 1). The HRs of mortality remained statistically significant for the low SES plus nonfrailty (*P* = 0.020), high SES plus frailty (*P* = 0.003), and low SES plus frailty group (*P* < 0.001) even after adjustments for age and sex (Model 1). The HR of the low SES plus frailty group still remained statistically significant (*P* = 0.015) even after additional adjustments for education, number of comorbidities, number of medications, alcohol consumption, smoking, nutritional status, disability, cognitive function, and fall experience (Model 2). However, the HRs of mortality were not statistically significant for the low SES plus nonfrailty (*P* = 0.107) and high SES plus frailty group (*P* = 0.069) when additionally adjusted for the covariates listed in Model 2.

In addition, we further explored any interactive and/or additive effect between low SES and frailty on all-cause mortality according to age-based subgroups (i.e., one aged 65–75 years and the other aged ≥76 years). In the 65–75-year group, the low SES plus nonfrailty group (HR = 1.56, 95% CI = 1.18–2.07, *P* = 0.002) and low SES plus frailty groups (HR = 2.50, 95% CI = 1.39–4.48, *P* = 0.002) had significantly higher all-cause mortality risks, as compared to the high SES plus nonfrailty group (referent, HR = 1). The HRs of the low SES plus nonfrailty and low SES plus frailty groups remained statistically significant (*P* = 0.038 and *P* = 0.021, resp.) even after adjustments for all the covariates. Yet, there was no statistically significant difference in all-cause mortality risk (*P* = 0.104) between the low SES plus nonfrailty and low SES plus frailty groups. Compared to the high SES plus nonfrailty group, the high SES plus frailty group had no significant difference (*P* = 0.316) in all-cause mortality risk. In the ≥76-year group, the high SES plus frailty and low SES plus frailty groups had significantly higher all-cause mortality risks (HR = 2.14, 95% CI = 1.32–3.47, *P* = 0.002 and HR = 2.00, 95% CI = 1.35–3.00, *P* = 0.001, resp.), as compared to the high SES plus nonfrailty group (HR = 1). The HRs of the two risk groups remained significant (*P* = 0.019 and *P* = 0.021, resp.) even after adjustments for age and sex but not statically significant (*P* = 0.228 and *P* = 0.222, resp.) when additionally adjusted for the covariates. Compared to the high SES plus nonfrailty group, the low SES plus nonfrailty group had no significant difference (*P* = 0.316) in all-cause mortality risk. Taken together, we found no interactive effect between the two exposures on all-cause mortality risk. Furthermore, the combined effects of low SES and frailty on mortality risk observed in the age-based subgroups were similar to that of the entire sample. Yet, the covariates were important confounders in determining the association among the older adults aged 76 years and older.

## 4. Discussion

In this population-based prospective study, we examined the combined effect of low SES and frailty on 3-year-all-cause mortality in Korean elderly persons aged 65 years and older. In this study, we found that frailty and low SES were associated with a number of covariates, including age, education, alcohol consumption, smoking, nutritional status, comorbidity, medication, dependent aging, cognitive function, and fall experience in this study population. In particular, this is the first study to report that a combination of low SES and frailty is an independent and additive determinant of increased all-cause mortality risk in Korean elderly persons.

The current findings of the study support and extend those of previous studies. Frailty has been well-established as an important predictor of all-cause and cause-specific mortality in older adults in Asian and Western countries. By analyzing data from the Living Profiles of Older People Survey involving 11,844 Koreans aged 65 years and older, Lee et al. [26] found that normal weight or underweight prefrail/frail status was an independent predictor of 3-year mortality in the Korean older population. Kim et al. [27] showed that a multidimensional frailty index was significantly associated with an increased risk of 1-year postoperative mortality in Korean geriatric patients. By analyzing data obtained from the InCHIANTI study involving 934 adults aged 65 years and older, Cesari et al. [28] showed that slow walking speed was an independent predictor of 6-year cumulative mortality in older adults in Chinese. In a population-based cohort involving 6724 women aged 69 years and older, Ensrud et al. [14] found that frailty was significantly associated with an increased risk of 9-year falls, fracture, and mortality independent of body mass. In addition, frailty was also associated with an increased mortality risk of older patients with colorectal cancers [29], elderly patients with myocardial infarction [30], patients with diabetes [31], and intensive care unit older patients [32], and chronic kidney disease patients [33].

Along with frailty, low SES is also known as another important predictor and/or modulator of all-cause and cause-specific mortality in older adults. In nationwide retrospective cohort study involving 1,025,340 Korean aged 65 years and older, Shin et al. [34] showed that femur-fracture patients with low and middle incomes had higher mortality rates than their counterparts with high income. In another population-based cohort study involving 11,946 patients with dyslipidemia aged 20 years and older, Shin et al. [35] showed that individuals living in less affluent neighborhoods were at significantly higher risk of all-cause mortality than individuals living in more affluent neighborhoods. In two cohort studies, Andrew et al. [36] investigated the association between social vulnerability and frailty and between social vulnerability and mortality in community-dwelling elderly persons in Canada. And they found that low SES was significantly associated with an increased mortality risk after adjustments for age, sex, and frailty. Additionally, SES was found to be significantly associated with an increased cause-specific mortality risk, including diabetes [37], cardiovascular disease [38], and prostate cancer [39]. Together, those findings including the current ones suggest that both low SES and frailty are important predictors of all-cause mortality in Korean older adults.

Finally, the combined effect of frailty and SES on all-cause mortality appears to be modulated by other covariates including age [40], number of comorbidities, lifestyle factors, and quality of life [41], and gender and community [42]. In regression models, therefore, this study included as many covariates as possible, including demographics (i.e., age, gender, and BMI), alcohol consumption and smoking, nutritional status, number of comorbidities and medications, disability, cognitive impairment, fall experience, and frailty. We found that compared with high SES plus nonfrailty, low SES or frailty alone or low SES plus frailty had a significantly increased all-cause mortality risk in Korean older adults, with no statistically significant differences among the risk groups, again suggesting that both low SES and frailty are important predictors of all-cause mortality rather than an additive and/or synergistic effect of the two risk factors.

Several explanations can be given for the increased risk of all-cause mortality associated with low SES and frailty. First, low SES and frailty are significantly associated with increased morbidities of chronic diseases such as type 2 diabetes [43], cardiovascular disease [44], and cancers [45], collectively contributing to an increased risk of all-cause mortality. Second, low SES and frailty also associate with mental disorder including depression, hopelessness, and social isolation, which may contribute to increasing the all-cause mortality [46, 47]. Third, lifestyle and dietary habit resulting in sarcopenia [48] and decreased cardiorespiratory fitness [49] may also contribute to an increased risk of death, because they are less likely to get a chance of medication. Lastly, frailty itself may be the end stage of a cascade of events from inflammatory processes to coagulative dysregulation to a range of alterations in hormones and peptides [50, 51] and thereby a threshold state characterized by increasing inability to adequately address physiological demands or a disruption of homeostatic mechanisms. Not surprisingly, the progressive accumulation of deficits secondary to frailty in conjunction with low SES would contribute to increased all-cause mortality risk in a cause-effect manner.

The present study had several strengths. Study participants of Korean older adults were recruited by stratified two-stage cluster sampling, and the overall response rates of 79.7% and 66.0% at baseline and at 3-year follow-up, respectively, were relatively high. In addition, the mortality data were gathered from a reliable register. As many covariates as possible were assessed in order to obtain a more reliable and reproducible association between the exposures and mortality. However, the study also had some limitations. First, the cross-sectional nature of this population-based prospective study does not allow for any causal inferences regarding the associations among low SES, frailty, and all-cause mortality. However, it is still possible that frailty may be an important factor in linking low SES to increased mortality risk [36] or low SES may be an important factor mediating the link between frailty and increased mortality risk [40]. Second, biomarker data, such as inflammatory cytokines, which might influence the relationships of low SES and frailty with mortality, were not available in this study. Third, the classification of SES into low or high based on the median value of household incomes of study participants may be biased and thereby weakens its representative of Korean older population. Fourth, although age was controlled as a covariate in our analysis due to limited sample size of old-old adults aged 85 years and older (i.e., approximately 4%), it may be possible that low SES differently modulates the association between frailty and all-cause mortality between old (aged 65~84 years) and old-old (aged 85 years and older) populations, as shown in the Chinese Longitudinal Healthy Longevity Survey [40]. Therefore, we cannot be certain that our findings would apply equally to old-old population in Korea. Fourth, it is known that parental SES has impacts on their SES in adulthood via childhood health status or vice versa [52]. Unfortunately, however, data regarding parental SES and their childhood health status were not obtained in this study. Therefore, a reverse causation between them and their impact on all-cause mortality later in life cannot be ruled out. Lastly, it is possible that the missing data at the follow-up (*N* = 576) might play a role, at least partially, in determining the association between frailty and all-cause mortality observed in this study because hospitalization or institutionalization is associated with frailty [53]. However, we failed to obtain any information regarding survival or death of the missing individuals at the 2011 follow-up, and this is another limitation.

## 5. Conclusion

In summary, the findings of this population-based prospective study suggest that either low SES or frailty is an independent predictor in determining increased all-cause mortality risk in Korean older adults, implying an urgency of implementing a public health policy targeting elderly persons with the two health disparities in Korea. The current findings also suggest that the relative contribution of low SES or frailty or a combination of the two risk factors to all-cause mortality risk remains to be further explored in a future study.

## Figures and Tables

**Table 1:** Baseline characteristics of study participants.

Measured parameters	Total (*N* = 7,960)	Men (*N* = 3,440)	Women (*N* = 4,520)	*P* value
Age (years)	72.2 ± 5.9	71.5 ± 5.5	72.8 ± 6.0	<0.001
BMI (kg/m^2^)	23.6 ± 3.2	23.2 ± 2.9	23.9 ± 3.4	<0.001
Household income (10,000 won)	143.5 ± 186.1	156.7 ± 188.7	133.4 ± 183.4	<0.001
Education, *n* (%)				<0.001
Uneducated	2,138 (30.9)	391 (13.1)	1,747 (44.4)	
Elementary school	2,604 (37.6)	1,107 (36.9)	1,497 (38.1)	
Middle-to-high school	1,678 (24.2)	1,092 (36.4)	586 (14.9)	
College or higher	508 (7.3)	406 (13.6)	103 (2.6)	
Frequency of weekly alcohol intake, *n* (%)				<0.001
<1	5,173 (74.7)	1,640 (54.8)	3,533 (89.8)	
1-2	1,196 (17.3)	859 (28.7)	337 (8.6)	
≥3	559 (8.1)	496 (16.6)	63 (1.6)	
Past/current smokers, *n* (%)	2,360 (34.1)	2,073 (69.2)	287 (7.3)	<0.001
Nutritional status (score)	3.2 ± 3.1	2.8 ± 2.9	3.5 ± 3.2	<0.001
Number of comorbidities	1.9 ± 1.5	1.5 ± 1.3	2.1 ± 1.6	<0.001
Number of medications	1.6 ± 1.3	1.3 ± 1.2	1.8 ± 1.3	<0.001
K-IADL (score)	10.7 ± 2.2	10.6 ± 2.3	10.8 ± 2.1	<0.001
MMSE-KC (score)	23.8 ± 4.2	25.3 ± 3.4	22.7 ± 4.3	<0.001
Fall experience, *n* (%)	1,055 (15.2)	333 (11.1)	722 (18.4)	<0.001
Frailty, *n* (%)				<0.001
Nonfrailty	6407 (92.6)	2,800 (93.8)	3,607 (91.7)	
Frailty	512 (7.4)	187 (6.2)	325 (8.3)	

BMI: body mass index, K-IADL: Korean version of instrumental activities of daily living, MMSE-KC: Mini-Mental State Examination in Korean of the CERAD assessment packet.

**Table 2:** Baseline characteristics of socioeconomic status (SES) and frailty-based subgroups.

	High SES plus nonfrailty (*N* = 3,781/47.5%)	Low SES plus nonfrailty (*N* = 3,556/44.7%)	High SES plus frailty (*N* = 227/2.9%)	Low SES plus frailty (*N* = 396/5.0%)	*P* value
Sex (% female)	1,934 (56.5)	1,673 (56.0)	141 (66.8)	184 (61.1)	0.008
Age (years)	71.2 ± 5.6	72.7 ± 5.7	76.3 ± 7.0	76.3 ± 6.4	<0.001
BMI (kg/m^2^)	23.7 ± 3.1	23.5 ± 3.3	23.4 ± 3.9	23.0 ± 3.6	<0.001
Household income (10,000 won)	232.1 ± 225.9	46.8 ± 22.5	221.5 ± 141.0	41.1 ± 21.6	<0.001
Education, *n* (%)					<0.001
Uneducated	782 (22.8)	1,093 (36.6)	91 (43.1)	171 (57.0)	
Elementary school	1,279 (37.3)	1,162 (38.9)	79 (37.4)	84 (28.0)	
Middle-to-high school	983 (28.7)	627 (21.0)	30 (14.2)	38 (12.7)	
College or higher	381 (11.1)	108 (3.6)	11 (5.2)	7 (2.3)	
Frequency of weekly alcohol intake, *n* (%)					<0.001
<1	2,557 (74.7)	2,182 (73.0)	180 (85.3)	254 (84.4)	
1-2	625 (18.2)	524 (17.5)	19 (9.0)	28 (9.3)	
≥3	243 (7.1)	285 (9.5)	12 (5.7)	19 (6.3)	
Past/current smokers, *n* (%)	1,111 (32.4)	1,082 (36.2)	57 (27.0)	110 (36.5)	0.001
Nutritional status (score)	2.3 ± 2.5	3.8 ± 3.2	4.6 ± 3.1	6.5 ± 3.6	<0.001
Number of comorbidities	1.8 ± 1.5	1.9 ± 1.5	2.5 ± 1.6	2.4 ± 1.6	<0.001
Number of medications	1.5 ± 1.3	1.6 ± 1.3	2.1 ± 1.3	2.2 ± 1.5	<0.001
K-IADL (score)	10.5 ± 1.7	10.6 ± 1.7	14.2 ± 5.4	12.4 ± 4.0	<0.001
MMSE-KC (score)	24.7 ± 3.8	23.4 ± 4.0	20.2 ± 5.2	20.0 ± 5.1	<0.001
Fall experience, *n* (%)	454 (13.3)	427 (14.3)	71 (33.6)	103 (34.2)	<0.001

SES: socioeconomic status; BMI: body mass index, K-IADL: Korean version of instrumental activities of daily living, MMSE-KC: Mini-Mental State Examination in Korean of the CERAD assessment packet.

**Table 3:** Combined effect of low SES and frailty on all-cause mortality risk.

	High SES plus nonfrailty	Low SES plus nonfrailty	*P* value	High SES plus frailty	*P* value	Low SES plus frailty	*P* value
		HR (95% CI)		HR (95% CI)		HR (95% CI)	
Total							
Model 0	Referent (HR = 1)	1.51 (1.23–1.85)	<0.001	2.86 (1.89–4.34)	<0.001	3.12 (2.26–4.28)	<0.001
Model 1	Referent (HR = 1)	1.28 (1.04–1.57)	0.020	1.88 (1.23–2.86)	0.003	1.85 (1.33–2.58)	<0.001
Model 2	Referent (HR = 1)	1.19 (0.96–1.47)	0.107	1.50 (0.97–2.33)	0.069	1.56 (1.09–2.23)	0.015

65–75 years							
Model 0	Referent (HR = 1)	1.56 (1.18–2.07)	0.002	1.59 (0.64–3.91)	0.316	2.50 (1.39–4.48)	0.002
Model 1	Referent (HR = 1)	1.48 (1.11–1.97)	0.008	1.67 (0.68–4.12)	0.267	2.41 (1.33–4.34)	0.004
Model 2	Referent (HR = 1)	1.37 (1.02–1.84)	0.038	1.47 (0.59–3.70)	0.416	2.09 (1.12–3.91)	0.021

76 years and older							
Model 0	Referent (HR = 1)	1.17 (0.87–1.57)	0.292	2.14 (1.32–3.47)	0.002	2.00 (1.35–3.00)	0.001
Model 1	Referent (HR = 1)	1.12 (0.84–1.51)	0.441	1.80 (1.10–2.91)	0.019	1.61 (1.08–2.41)	0.021
Model 2	Referent (HR = 1)	1.06 (0.78–1.43)	0.719	1.37 (0.82–2.29)	0.228	1.32 (0.85–2.06)	0.222

Model 0: unadjusted; Model 1: age and sex adjusted; Model 2: Model 1 + education, number of comorbidities, number of medications, alcohol consumption in frequency per week, smoking, nutritional status, K-IADL, MMSE-KC, and fall experience; SES: socioeconomic status; HR: hazard ratio; CI: confidence interval; K-IADL: Korean version of instrumental activities of daily living; MMSE-KC: Mini-Mental State Examination in Korean of the CERAD assessment packet.

## Supplementary Materials

Supplementary Materials Supplementary Table 1. Frailty criteria used in the study.
