# Supplementary material for: Socioeconomic Status, Frailty, and All-Cause Mortality in Korean Older Adults: A 3-Year Population-Based Prospective Study
Source: Biomed Res Int. 2017 Dec 14;2017:1903589. doi: 10.1155/2017/1903589 (PMC5745684; doi:10.1155/2017/1903589)
Supplement: Supplementary Materials — Supplementary Table 1. Frailty criteria used in the study. [file 1903589.f1.pdf]

Supplementary table 1. Frailty criteria used in the study

	Total, n (%)
Weight loss of >5kg	261 (3.8)
Exhaustion	1,627 (23.5)
Lowest 20% in BMI-specific hand-grip strength in this study population	1,337 (19.3)
Men:	
Strength ≤21.0kg for BMI ≤21.02kg/m^2	
Strength ≤23.0kg for BMI 21.03-22.98kg/m^2	
Strength ≤25.0kg for BMI 22.99-24.88kg/m^2	
Strength ≤25.5kg for BMI ≥24.89kg/m^2	
Women:	
Strength ≤13.0kg for BMI ≤21.67kg/m^2	
Strength ≤14.8kg for BMI 21.68-23.77kg/m^2	
Strength ≤15.0kg for BMI 23.78-25.97kg/m^2	
Strength ≤15.0kg for BMI ≥25.98kg/m^2	
Slowest 20% in height-specific gait speed	1,360 (19.6)
Men:	
≤0.49m/s for height ≤165.0cm	
≤0.50m/s for height ≥165.1cm	
Women:	
≤0.42m/s for height ≤151.9cm	
≤0.44m/s for height ≥152.0cm	
Lowest 20% in physical activity scale for the elderly	1,279 (18.5)
Men: ≤525.5kcal	
Women: ≤321.1kcal	

BMI: body mass index
